# Effects of External Potassium (K) Supply on Drought Tolerances of Two Contrasting Winter Wheat Cultivars

**DOI:** 10.1371/journal.pone.0069737

**Published:** 2013-07-10

**Authors:** Jiguang Wei, Caihong Li, Yong Li, Gaoming Jiang, Guanglei Cheng, Yanhai Zheng

**Affiliations:** 1 State Key Laboratory of Vegetation and Environmental Change, Institute of Botany, the Chinese Academy of Sciences, Beijing, PR China; 2 State Key Laboratory of Soil Erosion and Dryland Farming on the Loess Plateau, Northwest A&F University, Yangling, Shanxi, PR China; 3 University of the Chinese Academy of Sciences, Beijing, PR China; 4 Beijing Academy of Agriculture and Forestry Sciences, Beijing, PR China; University of Delhi South Campus, India

## Abstract

**Background:**

Drought is a common stress limiting crops growth and productivities worldwide. Water deficit may increase cellular membrane permeability, resulting in K outflow. Internal K starvation may disorder plant metabolism and limit plant growth. However, it is seldom reported about the effects of external K on drought tolerance of contrasting wheat cultivars.

**Methodology/Principal Findings:**

A hydroponics experiment was carried out in a non-controlled greenhouse. Seedlings of drought-tolerant SN16 and intolerant JM22 were simultaneously treated by five levels of K_2_CO_3_ (0, 2.5, 5, 7.5, 10 mM) and two levels of PEG6000 (0, 20%) for 7 days. External K_2_CO_3_ significantly increased shoot K^+^ content, water potential, chlorophyll content as well as gas exchange, but decreased electrolyte leakage (EL) and MDA content in both cultivars under PEG6000 stress. Antioxidant enzymes activities were up-regulated by PEG6000 while external K_2_CO_3_ reduced those changes. Molecular basis was explained by measuring the expression levels of antioxidant enzymes related genes. Shoot and root biomass were also increased by K_2_CO_3_ supply under drought stress. Although adequate K_2_CO_3_ application enhanced plant growth for both cultivars under drought stress, SN16 was better than JM22 due to its high drought tolerance.

**Conclusions/Significance:**

Adequate external K may effectively protect winter wheat from drought injuries. We conclude that drought-tolerant wheat combined with adequate external K supply may be a promising strategy for better growth in arid and semi-arid regions.

## Introduction

Potassium (K) is a key element for crops growth and productivities [1,2]. Normally, K^+^ influx is in balance with K^+^ outflow in K^+^-repletion plants, however, plant growth is limited if the K^+^ supply is interrupted, such as excessive K^+^ outflow caused by increasing of cellular membrane permeability [3–5]. Stress conditions (salinity, drought, etc.) influence the capacity of unidirectional K^+^ influx and net K^+^ uptake, while internal K^+^ concentration is exerted by both K^+^ influx and cell K^+^ conservation in the face of adversity [6].

Drought is a common stress to damage plant cellular membrane integrity [7,8]. Drought-induced overproduction of reactive oxygen species (ROS) may aggravate lipid peroxidation and increase cellular membrane permeability, resulting in excessive K^+^ outflow and limiting plant growth [9]. Ions outflow may increase the cytoplasm water potential and decrease plant water absorbance [10]. Internal K starvation may decrease some K^+^-depended enzymes activities and disorder plant physiological metabolism [11]. In addition, K starvation often activates overlapping cell signaling pathways and cellular responses, such as the reductions of chlorophyll production and photosynthetic capacity [12,13]. Electrolyte leakage (EL) and MDA content are two parameters to assess cellular membrane permeability and lipid peroxidation, and the increases of both parameters may indicate drought aggravation [5,10]. Antioxidant enzymes activities are direct indicators for ROS scavenging ability and plant drought tolerance [14,15].

Our previous studies show that external K may effectively elevate leaf K^+^ concentration of salinity-stressed wheat and protect winter wheat from salinity injuries [5,13]. However, little is known about the responses of drought-stressed winter wheat to exogenous K and the differential responses between cultivars with contrasting drought adaptation behaviors. Here, we hypothesize that external K may increase plant drought tolerance of winter wheat by enhancing internal K^+^ concentration under drought stress.

To test the above hypothesis, a hydroponics experiment was conducted to investigate the effects of exogenous K on leaf K^+^ content, water potential, chlorophyll content, gas exchange, cellular membrane integrity, antioxidant enzymes activities and shoot/root biomass yield of two winter wheat cultivars differing in drought tolerance. Molecular basis was also explained by measuring the expression levels of antioxidant enzymes related genes. More specifically, the present study addressed the following three questions: (1) Can external K significantly increase plant internal K and protect winter wheat from drought injuries? (2) Are there any differential responses to external K between wheat cultivars differing in drought tolerance? (3) What is the best combination between wheat variety and K application in arid and semi-arid regions?

## Results

### Gas exchange

**Figure 1:**
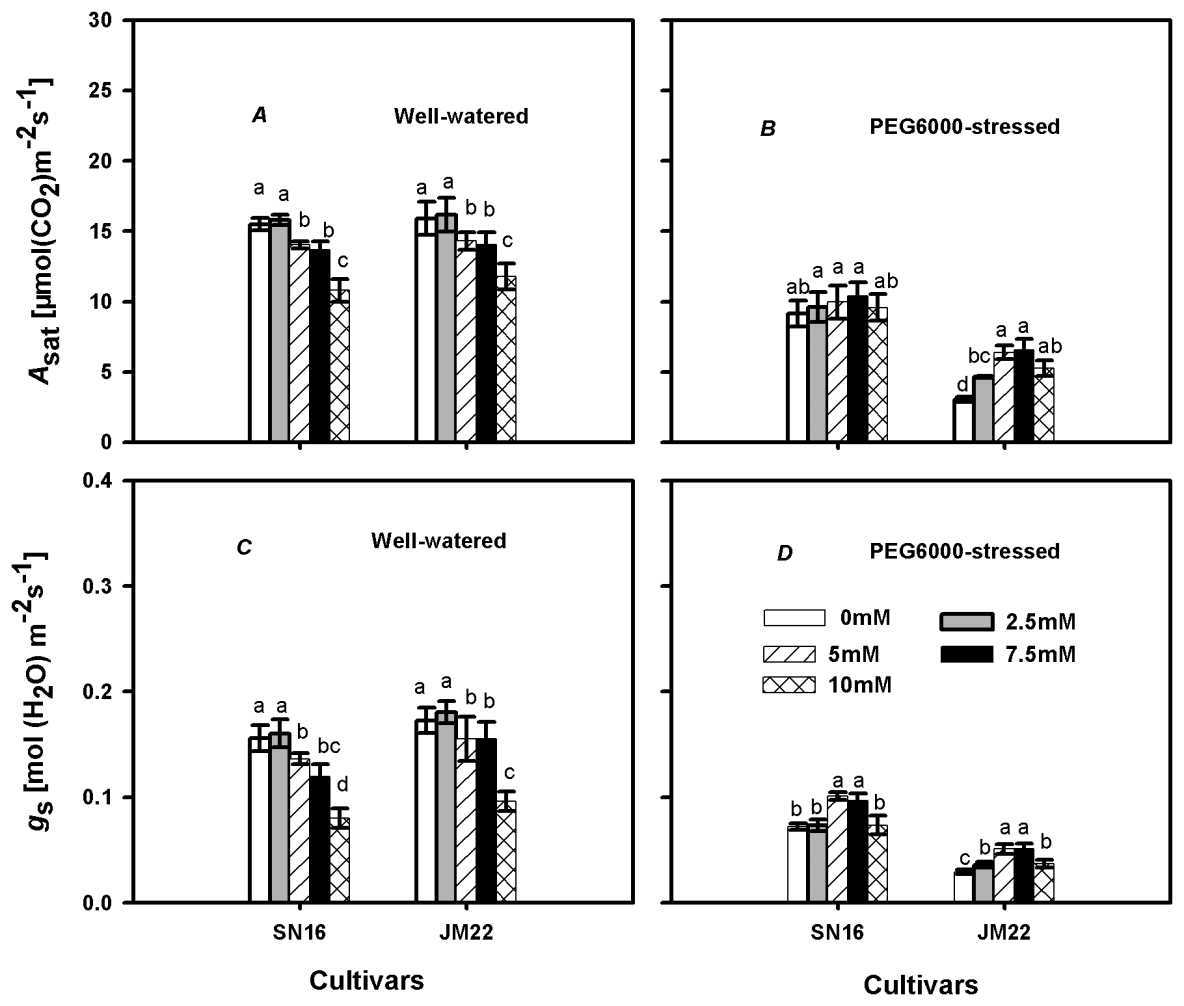
Effects of exogenous K_2_CO_3_ on light-saturated net photosynthetic rate (*A*_sat_) and stomatal conductance (*g*_s_) in SN16 and JM22 under different water treatments. (*A*, *C*) Plants are in well-watered condition. (*B*, *D*) Plants are in 20% PEG6000-stressed condition. Vertical bars denote SE (*n =6*). Different letters indicate significant at *p* ≤0.05.

In well-watered plants, non-significant effects of 2.5 mM K_2_CO_3_ concentration on light-saturated net photosynthetic rate (*A*_sat_) were noted in both cultivars, but significant adverse effects were measured at 5.0, 7.5 and 10.0 mM K_2_CO_3_ concentrations (Figure 1A). PEG6000 considerably reduced *A*_sat_ of both cultivars, with the extent being smaller in SN16 than in JM22 (Figure 1B). Under PEG6000-stressed condition, no significant increase of *A*_sat_ was found in SN16 but it did in JM22 by K_2_CO_3_ supply.

The variation trends of stomatal conductance (*g*_s_) were similar with *A*_sat_ in both cultivars in either well-watered condition (Figure 1C) or PEG6000-stressed condition (Figure 1D). Obviously, both *A*_sat_ and *g*_s_ were always higher in SN16 than in JM22 in each K_2_CO_3_ treatment under PEG6000-stressed condition.

### K^+^ contents in shoot and root

The K^+^ contents in both shoot and root were little higher in SN16 than in JM22 in control (no PEG6000, 0 mM K_2_CO_3_) plants (Table 1). External K_2_CO_3_ significantly increased internal K^+^ contents of both cultivars under well-watered conditions. PEG6000 drastically reduced K^+^ contents in both shoot and root of two cultivars. Adequate K_2_CO_3_ application increased K^+^ content in both shoot and root, with the extent being larger in root than in shoot in both cultivars. The K^+^ content in shoot peaked at 7.5 mM K_2_CO_3_ in both cultivars, while the maximum values in roots were noted in 10 mM K_2_CO_3_ treatment.

**Table 1:** Effects of external K_2_CO_3_ on shoot/root K^+^ content of drought-tolerant SN16 and intolerant JM22 under well-watered and PEG6000-stressed conditions.

Treatment			K^+^ content in SN16			K^+^ content in JM22	
PEG-6000 (w/v)	K_2_CO_3_ (mM)		Shoot (mg g^-1^ DM)	Root (mg g^-1^ DM)		Shoot (mg g^-1^ DM)	Root (mg g^-1^ DM)
0	0		43.2±0.7b	16.8±0.9d		40.7±0.8c	15.7±0.5d
	2.5		43.1±1.1b	21.8±0.7c		47.9±0.9b	20.4±0.7c
	5.0		43.5±0.9b	23.9±0.8b		51.1±1.2a	22.8±0.8b
	7.5		44.7±0.3a	25.8±0.6a		52.5±1.3a	26.6±0.9a
	10.0		47.0±1.4a	26.1±0.7a		53.5±1.4a	27.2±0.9a
20%	0		24.8±1.2b	8.9±0.2e		21.9±0.7c	8.7±0.1d
	2.5		28.5±2.1a	9.7±0.3d		24.5±0.8b	9.2±0.2c
	5.0		29.6±1.5a	10.5±0.3c		25.0±0.6a	10.0±0.2b
	7.5		30.6±1.1a	10.8±0.4b		25.8±0.5a	11.2±0.4a
	10.0		30.0±1.7a	11.7±0.3a		23.0±0.6b	11.7±0.6a

Significance analyses of data were performed using ANOVA analysis in SPSS package (Ver. 11, SPSS, Chicago, IL, USA). Within each column, means followed by different letter are significantly changed at *p* ≤0.05

### Water potential and chlorophyll content

**Figure 2:**
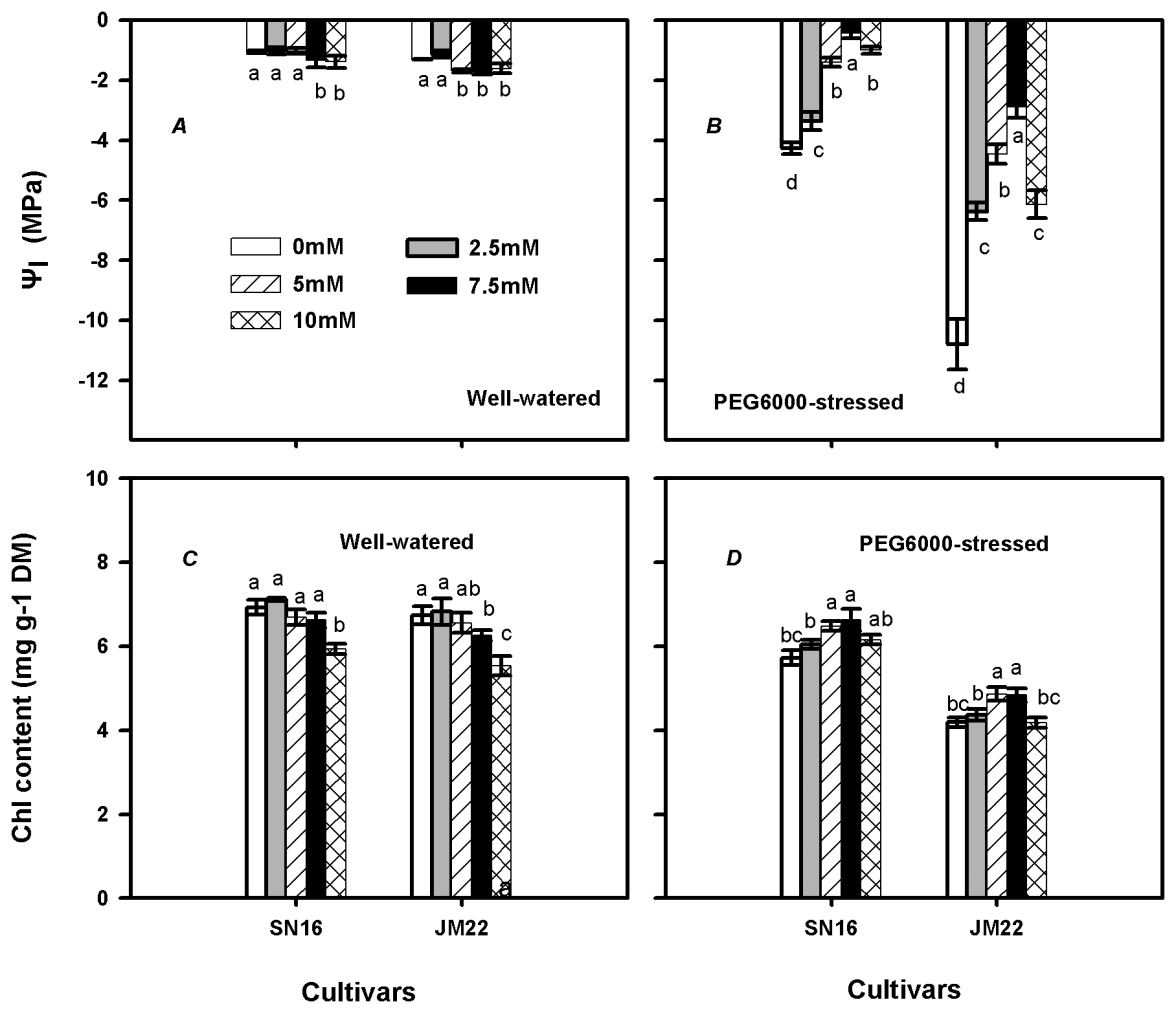
Effects of K_2_CO_3_ application on leaf water potential (*Ψ*_l_) and Chlorophyll (Chl) contents in SN16 and JM22 under different water treatments. (*A*, *C*) Plants are in well-watered condition. (*B*, *D*) Plants are in 20% PEG6000-stressed condition. Vertical bars denote SE (*n =6*). Different letters indicate significant at *p* ≤0.05.

Under well-watered condition, non-significant effects of 2.5 mM K_2_CO_3_ supply on Ψ_l_ were measured in both cultivars, while drastic decreases were noted in 7.5-10 mM K_2_CO_3_ treatments in SN16 and 5-10 mM K_2_CO_3_ treatments in JM22 (Figure 2A). PEG6000-induced reduction of Ψ_l_ was smaller in SN16 than in JM22. In PEG6000-stressed plants, adequate K_2_CO_3_ application significantly increased Ψ_l_ of both cultivars, and they peaked at 7.5 mM K_2_CO_3_ concentration (Figure 2B). The Ψ_l_ of SN16 was always higher than that of JM22 in each K_2_CO_3_ treatment under PEG6000-stressed condition.

In well-watered plants (Figure 2C), non-significant differences were determined in chlorophyll (Chl) contents of both cultivars in 2.5-7.5 mM K_2_CO_3_ application except 7.5 mM in JM22, however, considerable reductions occurred in 10 mM K_2_CO_3_ treatment than control. PEG6000 alone obviously decreased Chl contents, with the extent being smaller in SN16 than in JM22 (Figure 2C and D). Adequate external K_2_CO_3_ drastically increased Chl contents in both cultivars under PEG6000 stress.

### Electrolyte leakage and MDA content

**Figure 3:**
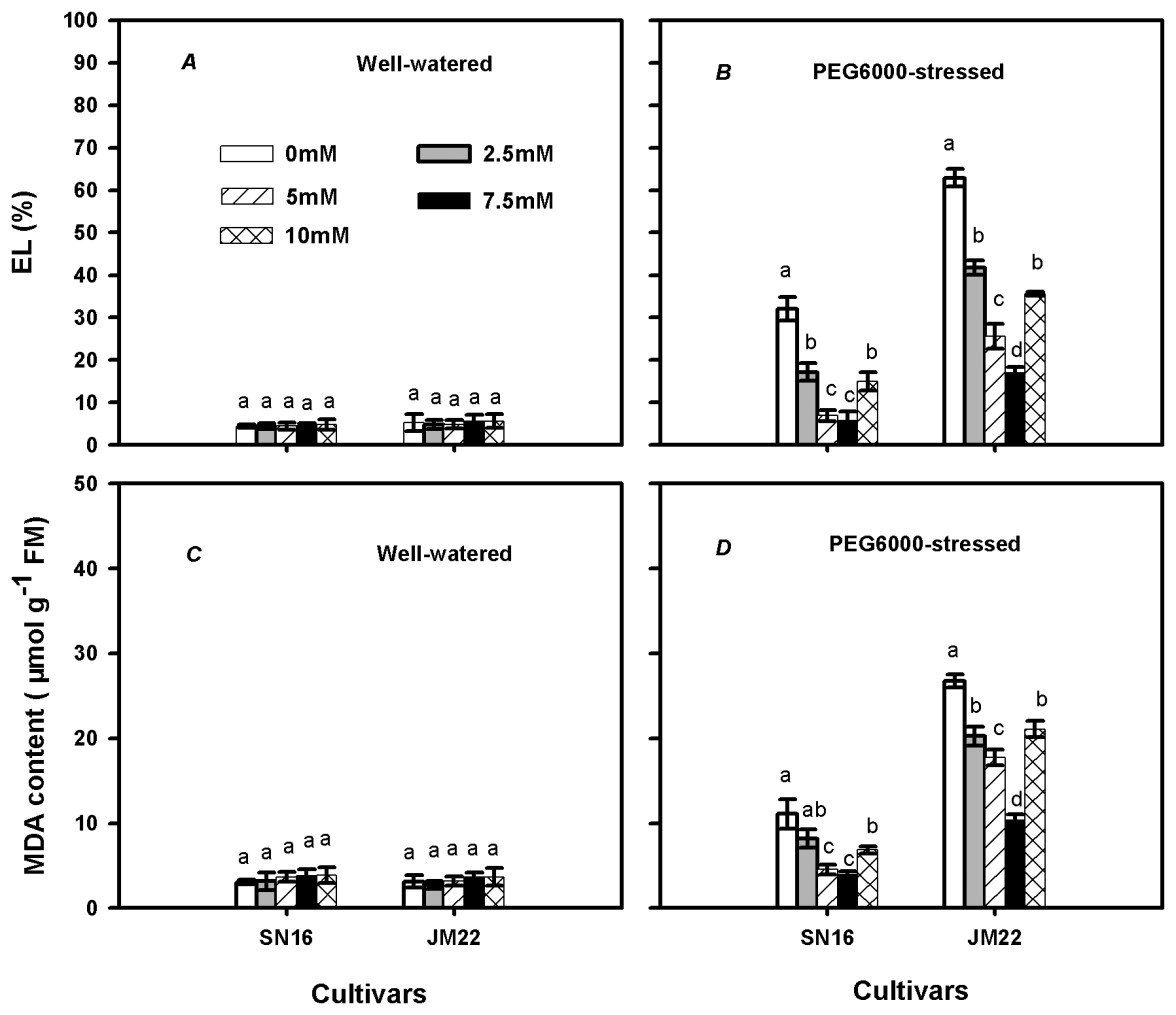
Effects of exogenous K_2_CO_3_ on electrolyte leakage (EL) and malondialdehyde (MDA) content in SN16 and JM22 under different water treatments. (*A*, *C*) Plants treated with 0% PEG6000. (*B*, *D*) Plants treated with 20% PEG6000. Vertical bars denote SE (*n =6*). Different letters indicate significant at *p* ≤0.05.

In well-watered plants, no significant differences were noted in electrolyte leakage (EL) between K_2_CO_3_ treatments in both cultivars (Figure 3A). PEG6000 significantly increased EL in both cultivars, with the extent being larger in JM22 than in SN16. Under PEG6000-stressed condition, external K_2_CO_3_ supply drastically reduced EL in both cultivars, with the best treatments being 5-7.5 mM for SN16 and 7.5 mM for JM22 (Figure 3B). However, higher EL was noted in 10 mM K_2_CO_3_ than in 7.5 mM K_2_CO_3_ treatment in both cultivars.

The responses of MDA content to K_2_CO_3_ application in both well-watered (Figure 3C) and PEG6000-stressed (Figure 3D) conditions were similar to the variation trends of EL in two wheat cultivars. Both EL and MDA content were smaller in SN16 than in JM22 in each K_2_CO_3_ treatment under PEG6000-stressed condition.

### Antioxidant enzymes activities and expression levels of their related genes

**Figure 4:**
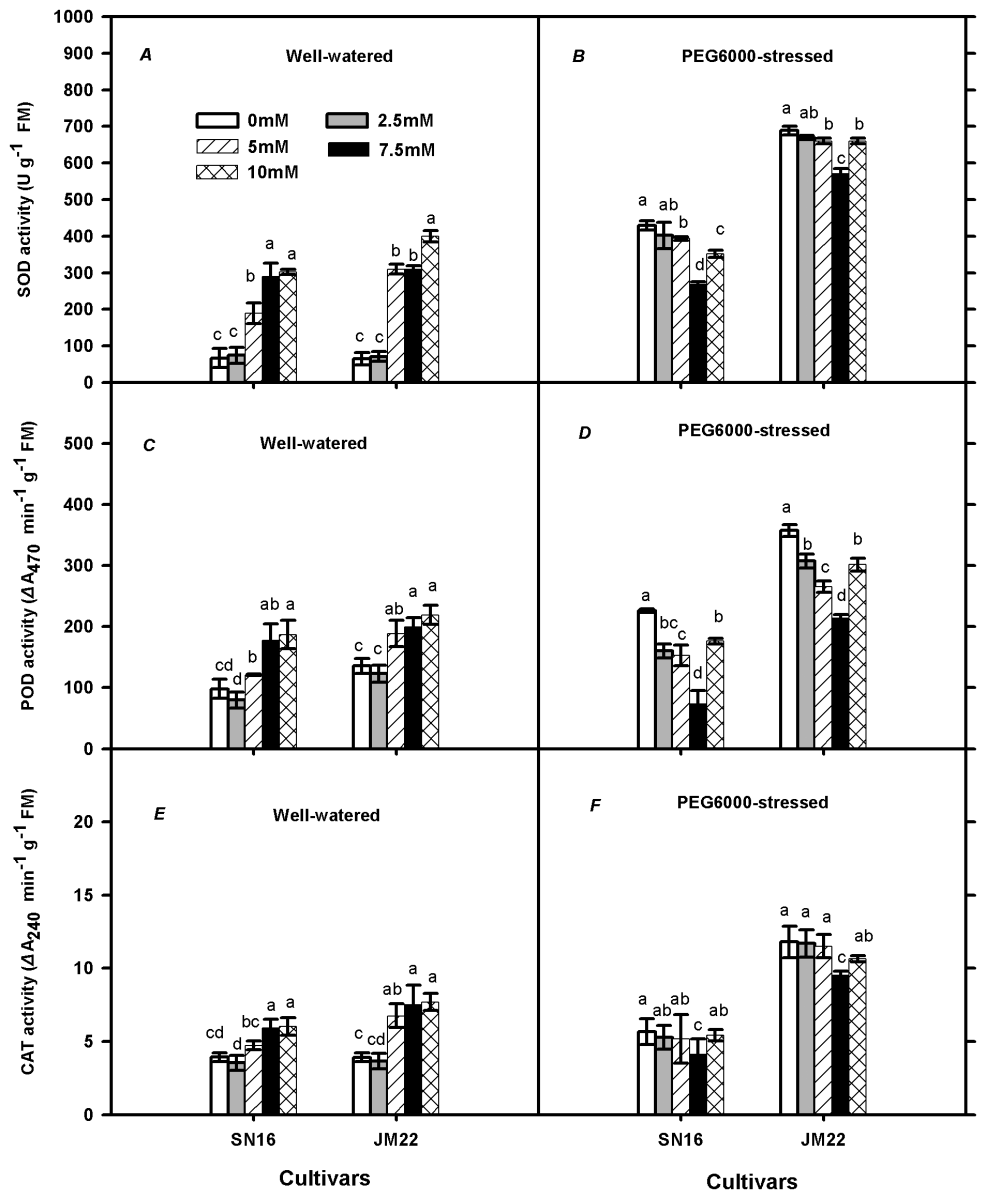
Effects of exogenous K_2_CO_3_ on superoxide dismutase (SOD), peroxidase (POD) and catalase (CAT) activities in SN16 and JM22 under different water treatments. (*A*, *C*, *E*) Plants treated with 0% PEG6000. (*B*, *D*, *F*) Plants treated with 20% PEG6000. Vertical bars denote SE (*n =6*). Different letters indicate significant at *p* ≤0.05.

In well-watered plants, superoxide dismutase (SOD) (Figure 4A), peroxidase (POD) (Figure 4C) and catalase (CAT) (Figure 4E) activities were significantly enhanced by K_2_CO_3_ application (except 2.5 mM) in both cultivars. PEG6000 increased those parameters in both cultivars, with the extents being larger in JM22 than in SN16. In PEG6000-stressed plants, only 7.5 mM external K supply drastically decreased the activities of SOD (Figure 4B) and CAT (Figure 4F), but significant effects of 2.5-7.5 mM K supply were noted on POD (Figure 4D) activities in both cultivars. Those antioxidant enzymes activities increased in 10 mM K_2_CO_3_ than in 7.5 mM K_2_CO_3_ treatment.

**Figure 5:**
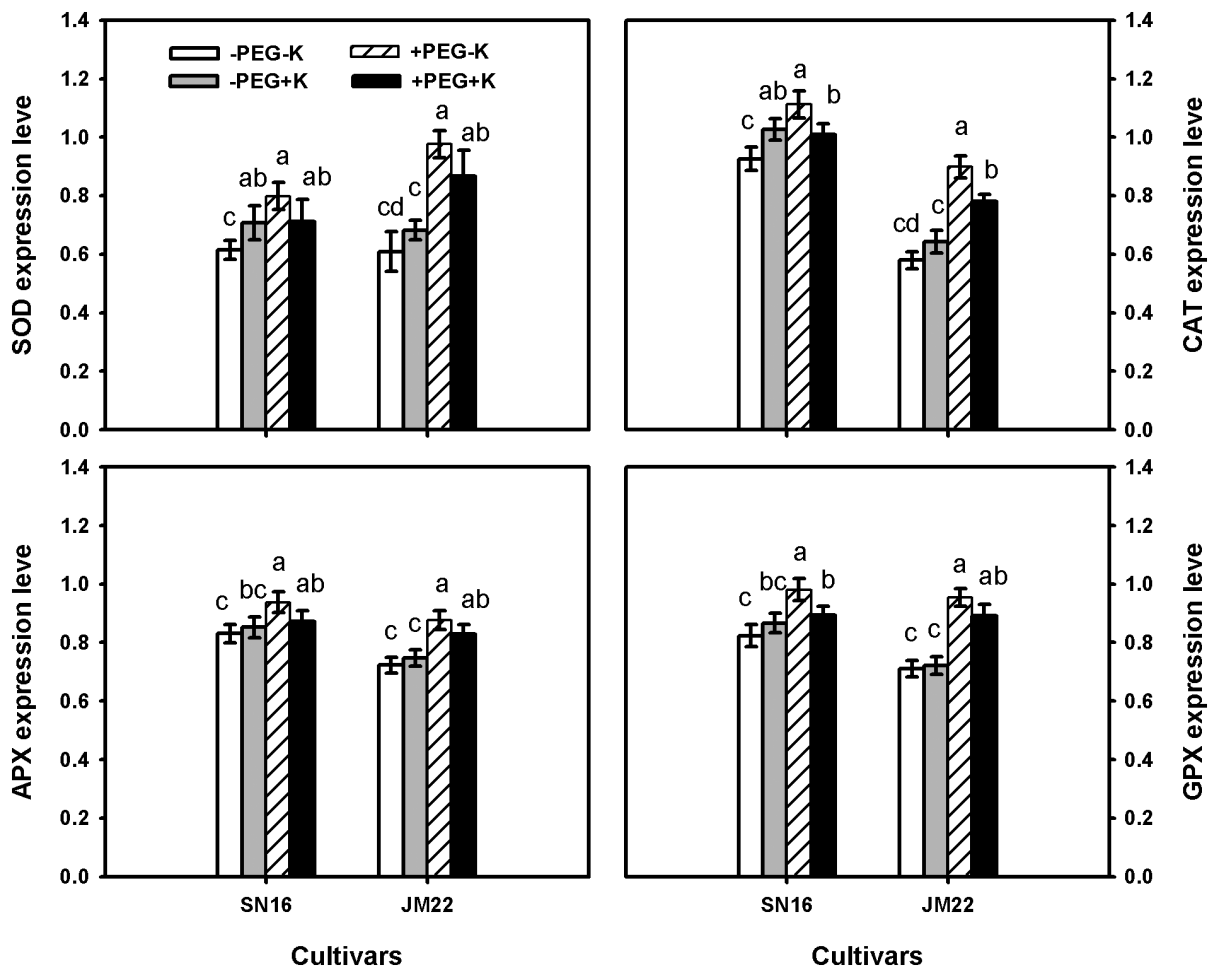
The expression levels of antioxidant enzymes related genes (SOD (*A*), CAT (*B*), APX (*C*) and GPX (*D*)) in SN16 and JM22 under different K_2_CO_3_ and PEG6000 treatments. Data are mean ± SE (*n* =3).

External K_2_CO_3_ (+K) supply significantly up-regulated the expression levels of SOD (Figure 5A) and CAT (Figure 5B) related genes of both SN16 and JM22 in –PEG but down-regulated them in +PEG condition. No-significant increases induced by K application were noted in the expression levels of APX (Figure 5C) and GPX (Figure 5D) related genes of two cultivars in –PEG but considerable decreases occurred in +PEG condition. Nevertheless, PEG alone (under –K condition) drastically up-regulated the expression levels of all antioxidant enzymes related genes in both cultivars.

### Plant growth and biomass yield

Plant height and root length peaked at 2.5 mM K_2_CO_3_ level in both cultivars in well-watered condition (Table 2). Both parameters were decreased by PEG6000 in two cultivars, with the extent being smaller in SN16 than in JM22. Under PEG6000-stressed condition, plant height and root length increased by adequate external K_2_CO_3_ and both parameters peaked at 5.0-7.5 mM in two cultivars.

**Table 2:** Effects of external K_2_CO_3_ on shoot/root dry masses of drought-tolerant SN16 and intolerant JM22 under well-watered and PEG6000-stressed conditions.

Treatment				Shoot			Root	
Cultivar	PEG6000 (w/v)	K_2_CO_3_ (mM)		Plant height (cm)	Dry mass (mg)		Root length (cm)	Dry mass (mg)
SN16	0	0		21.45±0.63b	0.31±0.03b		9.33±0.43a	0.11±0.00b
		2.5		23.45±0.93a	0.38±0.03a		10.02±0.92a	0.15±0.02a
		5.0		23.14±1.02a	0.36±0.02a		9.43±0.63a	0.14±0.03a
		7.5		22.02±0.82b	0.32±0.02b		9.41±0.71a	0.13±0.01a
		10.0		20.27±0.72c	0.27±0.02c		9.28±0.52a	0.10±0.01b
	20%	0		17.21±0.43b	0.22±0.03b		7.92±0.53b	0.09±0.00b
		2.5		17.83±0.52b	0.24±0.02b		8.21±0.72b	0.11±0.01a
		5.0		19.31±0.71a	0.29±0.02a		9.37±0.43a	0.13±0.02a
		7.5		20.01±0.93a	0.31±0.03a		9.39±0.63a	0.12±0.02a
		10.0		19.26±1.03a	0.26±0.02b		7.77±0.32b	0.10±0.01b
JM22	0	0		22.28±0.72b	0.28±0.02a		8.22±0.33b	0.08±0.00b
		2.5		23.92±0.64a	0.29±0.04a		9.21±0.73a	0.13±0.02a
		5.0		22.79±0.83b	0.29±0.02a		9.02±0.42a	0.12±0.02a
		7.5		21.28±1.03b	0.27±0.03a		8.59±0.62b	0.11±0.01a
		10.0		17.28±1.12c	0.23±0.03b		7.29±0.93c	0.06±0.01c
	20%	0		13.21±0.54b	0.19±0.04b		5.09±0.34b	0.07±0.00b
		2.5		14.10±0.63b	0.21±0.01b		5.28±0.56b	0.07±0.01b
		5.0		14.76±0.82a	0.26±0.02a		6.35±0.32a	0.09±0.01a
		7.5		14.15±0.55a	0.26±0.05a		6.12±0.55a	0.11±0.02a
		10.0		11.35±0.86c	0.23±0.06b		5.01±0.37b	0.08±0.01b

Significance analyses of data were performed using ANOVA analysis in SPSS package (Ver. 11, SPSS, Chicago, IL, USA). Within each column, means followed by different letter are significantly changed at *p* ≤0.05

Adequate K_2_CO_3_ application (2.5-5.0 mM) significantly increased shoot and root masses in both cultivars under well-watered condition. PEG6000 alone obviously decreased shoot and root dry masses in both cultivars. External K_2_CO_3_ significantly increased shoot and root masses of both cultivars under PEG6000-stressed condition, with peaks being noted in 5-7.5 mM K_2_CO_3_ treatment. The K_2_CO_3_-induced increases of biomass were larger in root than in shoot in both cultivars.

### PEG6000, K_2_CO_3_ and their interaction

Significant (*p* ≤0.05) effects of PEG6000, K_2_CO_3_ and PEG6000×K _2_CO_3_ (except root length) were noted on all growth, physiological and biochemical parameters (Table 3). Considerable effects of cultivar and cultivar×PEG6000 were also measured in most those parameters. However, no significant effects of cultivar×K_2_CO_3_ were noted in growth and physiological parameters except plant height. Cultivar×K_2_CO_3_×PEG6000 significantly affected most biochemical parameters, *A*_sat_, plant height and shoot mass, but not for root length and root mass.

**Table 3:** Effects (P-values) of cultivar, PEG6000, K_2_CO_3_ and their interactions on plant physiological and biochemical parameters of winter wheat analyzed using three-way (cultivar, PEG6000 and K_2_CO_3_ as the fixed factors) general linear model in SPSS (Ver. 11, SPSS, Chicago, IL, USA).

Parameters	Cultivars(Cv)	PEG6000(P)	K_2_CO_3_(K)	Cv×P	Cv×K	P×K	Cv×P×K
Plant height	**0.000**	**0.000**	**0.000**	**0.000**	**0.001**	**0.014**	**0.049**
Root length	**0.000**	**0.000**	**0.008**	**0.001**	0.914	0.840	0.786
Shoot mass	0.477	**0.000**	**0.000**	**0.042**	0.964	**0.000**	**0.025**
Root mass	**0.049**	**0.000**	**0.002**	**0.038**	0.392	**0.000**	0.398
*A*_sat_	0.062	**0.000**	**0.000**	**0.024**	0.263	**0.000**	**0.039**
*g*_s_	0.356	**0.000**	**0.000**	**0.001**	0.114	**0.000**	0.127
Shoot K^+^	0.379	**0.000**	**0.000**	**0.000**	**0.000**	**0.000**	**0.000**
Chl	**0.000**	**0.000**	**0.000**	**0.000**	**0.009**	**0.000**	**0.019**
*Ψ*_l_	**0.000**	**0.000**	**0.000**	**0.000**	**0.000**	**0.000**	**0.000**
EL	**0.000**	**0.000**	**0.000**	**0.000**	**0.000**	**0.000**	**0.000**
MDA	**0.000**	**0.000**	**0.000**	**0.000**	0.341	**0.000**	0.522
SOD	**0.000**	**0.000**	**0.000**	0.916	**0.020**	**0.000**	**0.000**
POD	**0.001**	**0.000**	**0.000**	**0.036**	**0.022**	**0.000**	**0.033**
CAT	**0.017**	**0.000**	**0.000**	0.173	0.102	**0.000**	**0.009**

*A*_sat_, light-saturated photosynthetic rate; *g*_s_, stamotal conductance; Chl, chlorophyll; EL, electrolyte leakage; MDA, malondialdehyde; SOD, superoxide dismutase; POD, peroxidase; CAT, catalase. The bold numerals highlight the significance at *p* ≤0.05.

## Discussion

### External K increased shoot/root K^+^ content and gas exchange

Stress-induced K^+^ outflow decreases internal K^+^ supply and limits plant growth [5,16,17]. Potassium starvation may cause plant physiological disorder, which lead to tissue dehydration in water-stressed crops [18–20]. In this study, adequate external K significantly increased K^+^ contents in both shoot and root of PEG6000-stressed plants (Table 1). This might be explained that (1) higher K^+^ concentration in plant growing medium offered more opportunity for roots absorbing K^+^; (2) cellular membrane recovery enhanced K^+^ conservation in plant tissues.

Stomatal conductance (*g*_s_) may be decided by the water potential which depends on ions concentration in stoma guard-cells [21–25]. External K might increase the K^+^ concentration in stoma guard-cells, leading to considerable enhancement of *g*_s_ [26–28]. Generally, significant positive correlation exists between *g*_s_ and light-saturated net photosynthetic rate (*A*_sat_) [3,13]. Drought-induced K^+^ outflow significantly decreased *g*_s_ and *A*_sat_ in both drought-tolerant and intolerant wheat, however, adequate K_2_CO_3_ application effectively recovered both parameters by increasing shoot/root K^+^ contents under drought stress. Both shoot and root biomasses were enhanced by adequate external K_2_CO_3_ supply in PEG6000-stressed condition.

### External K protected cellular membrane under drought stress

Drought-induced overproduction of reactive oxygen species (ROS) may aggravate cellular lipid peroxidation, leading to an increase of cellular membrane permeability, evidenced by increases of electronic leakage (EL) and MDA content [29–33]. High ions outflow may induce ions imbalance in plant tissues and lead to a physiological metabolic disorder [34–39]. In this study, adequate external K supply significantly reduced EL and MDA content (Figure 3), which might indicate that it protects cellular membrane of drought-stressed plants. This might be explained that (1) adequate K supply enhances cellular membrane stability [5,9]; (2) adequate external K supply may increase some solutes (such as proline, etc.) production and enhance plant osmotic adjustment ability [33]; (3) adequate external K reduces ROS production and its oxidative injuries under drought stress. However, excessive K supply also adversely affects the cellular membrane integrity in both drought-tolerant and intolerant winter wheat cultivars.

### Antioxidant enzymes activities and the expression levels of their related genes

Antioxidant enzymes activities were considered as indicators of scavenging ROS and reducing oxidative stress [18,20]. For example, SOD may convert superoxide radicals into H_2_O_2_ and H_2_O_2_ was further decomposed by CAT and POD [36]. In this study, PEG6000 significantly increased the antioxidant enzymes activities, with the extent being larger in drought-intolerant than in drought-tolerant wheat cultivar (Figure 4). Adequate external K supply significantly decreased antioxidant enzymes activities in PEG6000-stressed plants might be caused by enhancing plant physiological metabolism and reducing ROS production. However, excessive K supply also aggravated plant oxidative stress.

The expression levels of antioxidant enzymes related genes are known to be responsible for antioxidant enzymes production [18]. PEG6000 significantly up-regulated the expression levels of antioxidant enzymes related genes, with the extents being larger in drought-intolerant than in drought-tolerant wheat. Adequate external K_2_CO_3_ supply decreased the expression levels of antioxidant enzymes related genes in both cultivars under PEG6000-stressed condition. The results were consistent with the variation trends of antioxidant enzymes activities.

### Interaction of cultivar, PEG6000 and K_2_CO_3_ to plant growth

Significant (*P* ≤0.05) effects of cultivar, PEG6000 and K_2_CO_3_ were noted in growth, physiological and biochemical parameters (Table 3). The effects of cultivar were significant in plant height, root length and root mass. This might indicate that plant growth characteristics closely correlated with its drought tolerance. Significant effects of cultivar×PEG6000 were noted in growth parameters but not of cultivar×K_2_CO_3_. These might point out that cultivars differently response to PEG6000 but not to external K_2_CO_3_ supply. In addition, significant effects of PEG6000×K _2_CO_3_×cultivar were measured in plant height and shoot mass, which might indicate that significant interactions existed in aboveground plants between cultivar and external K supply under drought stress.

### Conclusions and future perspectives

Adequate external K supply protected winter wheat from drought injuries by reducing ROS production, enhancing K^+^ content and cellular membrane integrity, resulting in an increase of plant photosynthetic capacity.

Similar effects of external K were noted on drought-tolerant and intolerant wheat cultivars, however, the former growth performed better than the later due to its less damage by drought stress. Hence drought-tolerant cultivar combined with adequate external K supply may be a promising strategy for crops better growth and higher productivity in arid and semi-arid regions.

## Materials and Methods

### Plant culture

SN16, a hybrid of high-yield JN13 (female parent) × drought-tolerant 635 (male parent), is a typical drought-tolerant wheat cultivar which mainly demonstrated in arid and semi-arid croplands, and JM22, a drought-intolerant wheat cultivar, is a popular high-yield variety grown in adequate water farmlands in Shandong Province, China. Fifteen-day-old seedlings of SN16 and JM22 were equally transplanted and grown in 60 plastic boxes (length × width × height = 26 × 18 × 12 cm^3^) containing 1/2 Hoagland nutrient solution (pH =5.5), with 15 SN16 and 15 JM22 being separately planted in each box. Referring to our previous study on salt stress [5], five levels of K_2_CO_3_ (0, 2.5, 5, 7.5 and 10 mM) and two levels of PEG6000 (0, 20%) were simultaneously treated after 3 days adaptation. No K_2_CO_3_ and PEG6000 treatment was used as control. Six replications were exposed for each treatment. The pH value of each solution was adjusted to 5.5 with diluted H_2_SO_4_. Water lost was replenished daily by weighing method during the experiment. The photosynthetic photon flux density (PPFD) was approximately 800-1200 µmol m^-2^ s^-1^ at canopy height. The temperature in the greenhouse fluctuated from 15°C (night) to 25°C (day), with the relative humidity (RH) being kept in 15-35%. Physiological and biochemical parameters were measured 7 d after K_2_CO_3_ and PEG6000 treatments. And the expression patterns of antioxidant enzymes related genes were examined at transcriptional levels by qPCR in -K and +K (5 mM K_2_CO_3_) combined with –PEG and +PEG (20% PEG6000) treatments.

### Gas exchange

Gas exchange was measured on the most recently fully expanded leaves (third leaves) using a portable gas exchange system (*LI-6400, LI-COR*, USA). Relative humidity (RH) varied from 15–25%, and leaf temperature was set at 25 °C in the leaf chamber. The flow rate was set at 500 µmol s^-1^. The leaf was illuminated with a PPFD of 1200 µmol m^-2^ s^-1^ (saturated light) with an internal light source in the leaf chamber. After the gas exchange conditions were stabilized, the light-saturated net photosynthetic rate (*A*_sat_) and stomatal conductance (*g*_s_) were simultaneously recorded.

### Leaf water potential and chlorophyll content

Leaf water potential (Ψ_l_) was measured following the method of Jongdee et al. [40]. Ten most recently fully expanded leaves were sampled from 10 plants which grown in same box of each treatment, cut into pieces and spread evenly on the bottom of the sample cup to determine Ψ_l_ with a Water Potential Meter (*WP4-T*, *Decagon Devices*, Pullman, WA, USA).

Samples (0.05 g) were incubated in centrifuge tubes containing 10 ml 80% acetone under dark conditions for 36 h and centrifuged at 5000×*g* for 10 min. The extracts were decanted and completed to 25 ml. The absorbance of the extracts was recorded at 663 and 645 nm, respectively. Chlorophyll contents were calculated using the following formulas [30]:

Chl (mg g^-1^ DM) =(20.2×*A*_645_+8.02×*A*_663_) ×*V*/(*DM* × 1000)

Where *A*_663_ and *A*_645_ are the absorbance at 663 and 645 nm, respectively, *V* is the volume of the extract and *DM* is the dry mass of the sample.

### K^+^ contents

K^+^ contents in shoot and root of winter wheat were assayed following the method described by Zheng et al. [13]. Dry samples of shoots and roots were finely ground before passing through a 2-mm sieve. Approximately 0.2 g samples were soaked for 12 h in digesting tubes with 8 ml concentrated nitric acid and 2 ml perchloric acid and then digested at 300°C for 6 h. The extractions were completed to 50 ml with deionized water. K^+^ contents were determined using a sequential plasma spectrometer (*ICPS-7500*, *SHIMADZU*, JAPAN).

### Cellular membrane permeability

Cellular membrane permeability was assessed by measuring electrolyte leakage (EL) following the method described by Tripathy et al. [41]. Ten 4-cm length leaf middle sections were soaked in test tubes containing 10 ml deionized water and incubated at 25°C for 2 h to measure the initial electrical conductivity (EC_1_). The samples were autoclaved at 100°C for 20 min to release electrolytes, and then cooled to 25°C to measure the final electrical conductivity (EC_2_). EL was calculated using the following formula:

EL = 100×EC_1_/EC_2_

### Lipid peroxidation and antioxidant enzyme activities

Leaf samples were collected, immediately frozen in liquid nitrogen and transferred to an ultra-freezer at -80 °C until the time of assay. Samples (about 0.5 g) were crushed into fine powder in an ice-cold mortar with 10 ml 0.05 M KH_2_PO_4_ (pH 7.8) buffer. The homogenate was centrifuged at 10000×*g* for 20 min, with the supernatants being further prepared to measure MDA contents and antioxidant enzyme activities. These processes were performed at 0–4 °C.

Lipid peroxidation was assessed by measuring the malondialdehyde (MDA) content according to Draper and Hadley [33]. A mixture of 1 ml extract and 2 ml 0.6% thiobarbituric acid (TBA) was boiled for 15 min, cooled and centrifuged for 10 min at 10000×*g*. The content of MDA was determined from the absorbance at 600, 532 and 450 nm and then calculated using the following formula:

MDA (μmol g^-1^ FM) = (6.45×(*A*_532_-*A*_600_)-0.56×*A*_450_) ×*V*_t_/(*V*_s_×FM)

where *A*_600_, *A*_532_ and *A*_450_ are the absorbances at 600, 532 and 450 nm, respectively, *V*_t_ is the volume of extract, *V*_s_ is the volume of the test extract and FM is the fresh mass of the sample.

Superoxide dismutase (SOD) activity was determined following the method described by Zheng et al. [13]. One enzyme unit of SOD activity (U) was defined as the amount of crude enzyme extract required for inhibiting the reduction rate of nitro-blue tetrazolium (NBT) by 50%. Peroxidase (POD) was estimated by measuring the oxidation of guaiacol. The guaiacol reagent was composed of 50 ml 0.1 M KH_2_PO_4_ (pH 6.0) buffer, 28 µL guaiacol and 19 µL 30% H_2_O_2_. A mixture of 20 µL crude enzyme extract + 3 ml guaiacol reagent was formed, and the absorbance was continuously recorded five times at 470 nm in 30 s intervals. Variation of absorbance per minute per gram fresh mass (⊿A470 min-1 g-1 FM) served for POD activity. Activity for CAT was expressed as variation of absorbance per minute per gram fresh mass (⊿A240 min-1 g-1 FM). All spectrophotometric analyses were conducted on a UV/visible light spectrophotometer (*UV-2450, SHIMADZU*, JAPAN).

### Plant growth and biomass yield

Plants were harvested immediately after the above physiological and biochemical measurements. Twenty plants of individual cultivar which randomly picked from six replicates of each K_2_CO_3_ and PEG6000 treatments were cleaned and measured plant height and root length, then separated into shoots and roots and oven-dried at 75°C to constant weight. Biomass of shoot and root was then recorded, respectively.

### Expression patterns of antioxidant enzymes related genes

Total RNAs were extracted from harvested leaves of wheat with Trizol reagent (Invitrogen). The total RNAs were reversely transcribed into first-strand cDNA with PrimeScript^®^ RT reagent Kit With gDNA Eraser (Takara Bio), and the cDNAs obtained were used as templates for PCR amplification with specific primers. Gene-specific primers used for RT-qPCR were: 5’-TTG TAG GTC GCT GGT TTC-3’ and 5’-CCA AGT TCA CGG TTC ATA G-3’ for *TaSOD* (U69536.1); 5’-AGT TGG ACG GAT GGT ACT GA-3’ and 5’-AAG ACG GTG CCT TTG GGT-3’ for *TaCAT* (X94352.1); 5’-GAC GGC TGA ATG GTT GAA-3’ and 5’-AAT GCC TCC TGG TCC TCT-3’ for *TaAPX* (AF387739.1); 5’-AAC TAC CCG CTC TGC TCC T-3’ and 5’-GCC TTG GTC CTT GTA CTT CG-3’ for *TaGPX* (AJ010455.1). In addition, *TaActin* (AB181991.1) was used as internal control: 5’-CTA TCC TTC GTT TGG ACC TT-3’ and 5’-AGC GAG CTT CTC CTT TAT GT-3’. RT-qPCR was performed using StepOne instrument. Each reaction contained 10 µl 2×SYBR Green Master Mix reagent (Takara Bio), 1.0 µl cDNA samples, 1.2 µl 10 mM gene-specific primers and 0.4 µl 50×ROX in a final volume of 20 µl. The thermal cycle was used as followings: 95°C for 2 min; 40 cycles of 95°C for 30s, 55°C for 30s, and 72°C for 30s. The expression levels of antioxidant enzymes were analyzed by the comparative Ct method.

### Statistical analysis

Significance analyses of data were performed for each cultivar under each condition using ANOVA analysis in SPSS package (Ver. 11, SPSS, Chicago, IL, USA). The effects of Cultivar, PEG6000, K_2_CO_3_ and their interactions were analyzed using three-way (cultivar, PEG6000 and K_2_CO_3_ as the fixed factors) general linear model on each measured dependent variable. The significance was considered at *p* ≤0.05.
